# Pretreatment resistance mutations and treatment outcomes in adults living with HIV-1: a cohort study in urban Malawi

**DOI:** 10.1186/s12981-020-00282-3

**Published:** 2020-05-20

**Authors:** F. Neuhann, A. de Forest, E. Heger, A. Nhlema, C. Scheller, R. Kaiser, H. M. Steffen, H. Tweya, G. Fätkenheuer, S. Phiri

**Affiliations:** 1 Heidelberg Institute for Global Health, University Hospital of Heidelberg, Im Neuenheimer Feld 130.3, 69120 Heidelberg, Germany; 2 Institute of Virology, University of Cologne, Faculty of Medicine and University Hospital of Cologne, Cologne, Germany; 3 Lighthouse Clinic, Lilongwe, Malawi; 4 Institute of Virology and Immunobiology, University of Würzburg, Würzburg, Germany; 5 Clinic for Gastroenterology and Hepatology, Faculty of Medicine and University Hospital of Cologne, Cologne, Germany; 6 Department of Internal Medicine I, Faculty of Medicine and University Hospital of Cologne, Cologne, Germany; 7 Department of Global Health, University of Washington, Seattle, WA 98104 USA; 8 Department of Medicine, University of North Carolina School of Medicine, Chapel Hill, NC USA; 9 Department of Public Health, College of Medicine, School of Public Health and Family Medicine, University of Malawi, Zomba, Malawi

**Keywords:** HIV, Drug resistance mutations, Pretreatment resistance, Non-nucleoside reverse transcriptase, Malawi

## Abstract

**Background:**

Pre-treatment drug resistance (PDR) among antiretroviral drug-naïve people living with HIV (PLHIV) represents an important indicator for the risk of treatment failure and the spread of drug resistant HIV variants. We assessed the prevalence of PDR and treatment outcomes among adults living with HIV-1 in Lilongwe, Malawi.

**Methods:**

We selected 200 participants at random from the Lighthouse Tenofovir Cohort Study (LighTen). Serum samples were drawn prior to treatment initiation in 2014 and 2015, frozen, and later analyzed for the presence of HIV-1 drug resistance mutations. Amplicons were sequenced and interpreted by Stanford HIVdb interpretation algorithm 8.4. We assessed treatment outcomes by evaluating clinical outcome and viral suppression at the end of the follow-up period in October 2019.

**Results:**

PDR testing was successful in 197 of 200 samples. The overall NNRTI- PDR prevalence was 13.7% (27/197). The prevalence of intermediate or high level NNRTI- PDR was 11.2% (22/197). The most common mutation was K103N (5.6%, 11/197), followed by Y181C (3.6%, 7/197). In one case, we detected an NRTI resistance mutation (M184V), in combination with multiple NNRTI resistance mutations. All HIV-1 isolates analyzed were of subtype C. Of the 27 patients with NNRTI- PDR, 9 were still alive, on ART, and virally suppressed at the end of follow-up.

**Conclusion:**

The prevalence of NNRTI- PDR was above the critical level of 10% suggested by the Global Action Plan on HIV Drug Resistance. The distribution of drug resistance mutations was similar to that seen in previous studies from the region, and further supports the introduction of integrase inhibitors in first-line treatment in Malawi. Furthermore, our findings underline the need for continued PDR surveillance and pharmacovigilance in Sub-Saharan Africa.

## Introduction

The global availability of antiretroviral therapy (ART) has resulted in a great reduction of new HIV infections, HIV related morbidity, and mortality [1, 2]. However, settings with the highest prevalence of HIV often lack critical resources, including infrastructure for monitoring the development of HIV drug resistance mutations. In low-income countries, drug resistance surveillance is only performed periodically at specific sites or in populations under treatment, and rarely prior to treatment initiation. The development and spread of HIV drug resistance (HIVDR) could endanger treatment success, and ultimately threaten the control of the epidemic [3].

Pretreatment drug resistance (PDR) is defined by the WHO as resistance that is detected among people either newly initiating or reinitiating first-line ART [3]. In previous studies, non-nucleoside reverse transcriptase inhibitor (NNRTI) related PDR exceeded 10% in many African settings, supporting the move to include the integrase inhibitor dolutegravir (DTG) in first-line regimens [4, 5].

Malawi has set up an effective HIV treatment program. According to the Malawian Ministry of Health quarterly reports, as of September 2018, out of the total one million PLHIV in Malawi, around 94% knew their status, 80% were on ART, and 89% of those on ART were virally suppressed [6].

The Lighthouse Clinic is the largest provider of HIV counselling, treatment and care in Lilongwe, Malawi [7]. We commenced the Lighthouse Tenofovir Cohort Study (LighTen; ClinicalTrials.gov NCT02381275) in August 2014. We enrolled 1432 ART-naïve adults living with HIV and followed them from initiation with tenofovir-based ART for a period of 36 months, with the primary objective of analyzing changes in kidney function. At the onset of the study, the Lighthouse clinic was providing comprehensive HIV services to over 24 000 PLHIV in greater Lilongwe (own data). We report the frequency and pattern of pretreatment HIV-1 drug resistance (PDR) and the treatment outcomes among a subgroup of this cohort.

## Methods

### Setting

The study was conducted at one of the Lighthouse clinics on the campus of the Kamuzu Central Hospital in Lilongwe, Malawi. The Lighthouse is a large, specialized center for HIV treatment and care in the central region of Malawi, and mainly serves an urban population of lower socio-economic status. Access to testing and counselling as well as treatment and care is free of charge at the point of delivery [7].

### Participants

The present study is a subgroup analysis of participants in the LighTen study, which enrolled 1432 ART-naïve adults aged 18 years or older. 200 consecutively enrolled patients were selected at random for PDR testing during the recruitment process in 2014 and 2015. We drew baseline serum samples prior to treatment initiation, stored them at minus 80° C, and later analyzed them for the presence of HIV-1 drug resistance mutations.

All LighTen enrollees received first-line ART according to Malawian treatment guidelines, which at the time of enrolment consisted of lamivudine (3TC), tenofovir disoproxil fumarate (TDF) and efavirenz (EFV). Decisions on treatment regimen and potential switches were not informed by the results of drug resistance testing. Participants were seen regularly during routine follow-up visits as scheduled in the Malawian national treatment guidelines and according to clinical needs. Treatment outcomes were analyzed at the end of 2019.

### Laboratory processes

All participants had baseline evaluation beyond the standards of the Malawian HIV treatment program, including full blood count, liver and renal function tests, CD4 cell count, and HIV viral load testing (see LighTen protocol; ClinicalTrials.gov NCT02381275).

Nucleic acid extraction from 500 µL of serum was performed using the DNA and viral NA large volume kit (Roche Diagnostics, Mannheim, Germany) for the automated MagNA Pure 96 system (Roche Diagnostics, Rotkreuz, Switzerland). The protease and reverse transcriptase regions were amplified for resistance analysis as described by Lübke et al. [8], and the envelope region for tropism determination as described by Sierra et al. [9]. For HIV subtyping, the COMET tool version 2.2 [10] was used. Amplicons were sequenced by Next Generation Sequencing using Illumina Sequencing Technology (Illumina Inc., San Diego, USA) and interpreted by Stanford HIV database interpretation algorithm 8.4 (HIVdb) [11].

### Outcomes

Outcome variables were: viral load 6 months after treatment initiation, last viral load, and treatment outcome at the time of assessment. Treatment outcomes were categorized as either: alive and on treatment at the Lighthouse clinic, transferred out to another treatment facility, stopped ART, died, changed ART regimen, withdrawn from the study, or defaulted. The status “defaulted” was assigned to patients who had not been in contact with the clinic 60 days after a missed follow-up appointment. Drug resistance mutations conferring at least low-level resistance according to the Stanford HIVdb [9] were counted, and the treatment outcomes of affected patients were analyzed.

### Statistical methods

We compared the group included in drug resistance testing to the cohort not included in resistance testing at baseline using Chi square, Student’s Test and Kruskall-Wallis test as appropriate for the type of variable and the respective distribution. The significance level was set at 0.05.

### Ethics

The LighTen study protocol was approved by the Ethics Committee of the National Health Research Committee of the Ministry of Health, Malawi and the ethics committees of the Universities of Heidelberg and Cologne, Germany.

## Results

Overall, LighTen enrolled 1432 participants, of whom 200 were included in the HIV-1 resistance testing group. HIVDR testing was successful in 197 of 200 samples. All analyzed HIV-1 isolates were of subtype C.

Baseline characteristics of the HIVDR testing group differed from the total LighTen cohort, with a higher proportion of WHO stage 1 (65% vs 43%) and a lower proportion of WHO stage 3 (9.6% vs 34.7%) in the HIVDR testing group. The HIVDR testing group had a higher median viral load. The groups also differed significantly regarding the treatment outcome “Alive and on ART” (Table 1).

**Table 1:** Comparison of participants with and without HIVDR testing

	Participants		P
	No HIVDR testing	HIVDR testing	
n	1235	197	
Sex (%)			
Female	692 (56.0)	122 (61.9)	0.140^a^
Male	543 (44.0)	75 (38.1)	
Age [mean (SD)]	36.20 (9.31)	35.09 (9.11)	0.118^b^
BMI [mean (SD)]	24.28 (4.91)	23.93 (4.63)	0.348^b^
WHO stage (%)			
1	531 (43.0)	128 (65.0)	< 0.001^a^
2	183 (14.8)	38 (19.3)	
3	429 (34.7)	19 (9.6)	
4	92 (7.4)	12 (6.1)	
CD4 count (median [IQR])	269.5 [125; 420]	247.5 [89; 420]	0.230^c^
Viral load (median [IQR])	33 000 [6 696; 140 844]	112 599 [21 318; 454 638]	< 0.001^c^
Outcome (%)			
Alive on ART	715 (57.9)	119 (60.4)	0.020^a^
Defaulted	267 (21.6)	32 (16.2)	
Transferred out	118 (9.6)	21 (10.7)	
Changed ART regimen	62 (5.0)	3 (1.5)	
Died	41 (3.3)	14 (7.1)	
Withdrawn	27 (2.2)	7 (3.6)	
Stopped ART	5 (0.4)	1 (0.5)	

CD4 count: number of CD-4 positive T-cells per µl; viral load: number of copies of HIV-1 RNA per ml of Serum

*n* number of participants, *SD* standard deviation, *IQR* interquartile range, *HIVDR* HIV-1 drug resistance mutation, *BMI* body mass index, kg/m^2^, *WHO stage* World Health Organization stage of clinical HIV illness

^a^Chi^2^ test; ^b^Student’s *t* test; ^c^Kruskal Wallis test

The overall NNRTI-PDR prevalence was 13.7% (27/197). The prevalence of mutations conferring intermediate or high level resistance to first-line ART was 11.2% (22/197). The most common PDR was K103N (5.6%, 11/197), followed by Y181C (3.6%, 7/197). In one case, we detected an additional NRTI drug resistance mutation (M184V) (Table 2). We identified the accessory mutation E138A in eight samples.

**Table 2:** Overview and frequency of identified mutations in 27 patients (potentially treatment relevant mutations in italic)

Identified mutations (n)			
NRTI	Accessory PI	NNRTI	Resistance level to EFV^a^
*M184 (1)*	T47S (7)	*K103N (11)*	High
	Q58E (4)	*A98G (3)*	High level resistance *3TC*, Low level resistance EFV
	K20T (1)	*V106M (2)*	High
	M46L (1)	*Y181C (7)*	Intermediate
	M46V (1)	*G190A (2)*	Intermediate
	N88D (1)	*K103S (1)*	Intermediate
		*K238T (1)*	Intermediate
		H221Y (4)	Low
		V108I (4)	Low
		E138G (2)	Low
		V179D (2)	Low
		E138K (1)	Low
		K101E (1)	Low

The total number of mutations reported is higher than the number of individual samples with NNRTI-DRMs, as many samples showed multiple mutations

*NRTI* nucleoside/nucleotide reverse transcriptase inhibitors, *PI* protease inhibitors, *NNRTI* non-nucleoside reverse-transcriptase inhibitors, *EFV* efavirenz, *3TC* lamivudine

^a^According to the Stanford Drug Resistance Database

**Fig. 1:**
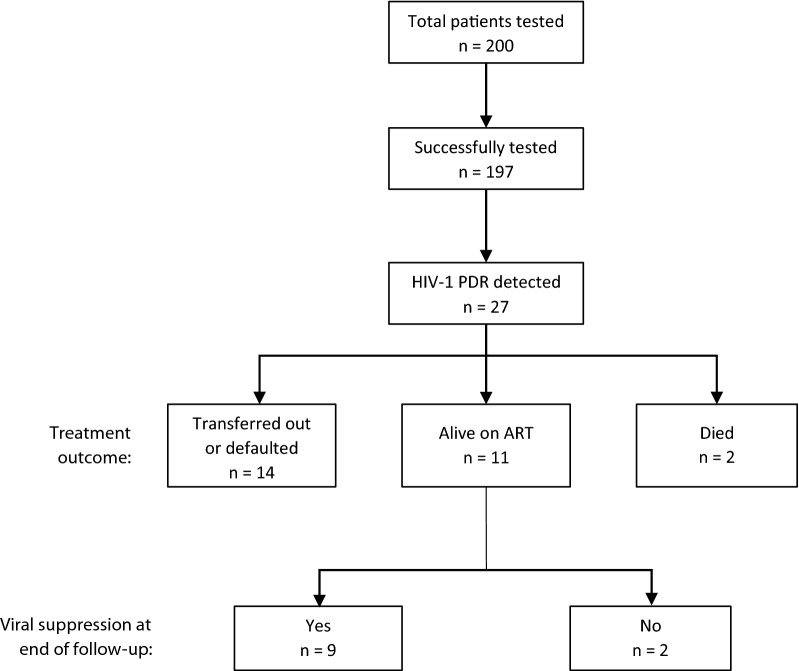
Flowchart of HIV-1 pretreatment drug resistance testing and treatment outcomes. *PDR* HIV-1 pretreatment drug resistance, *ART* antiretroviral therapy. For details on treatment outcome categories, see text

Of the 27 individuals with NNRTI- PDR mutations, 11 were still alive and on treatment at Lighthouse at the end of follow-up, 9 of whom were virally suppressed. Of the 16 patients not alive and on treatment at the end of follow-up, 12 had defaulted, two had transferred to another clinic and two had died (see Fig. 1). A synopsis of the treatment outcomes is provided in Table 3.

**Table 3:** Synopsis of baseline characteristics and clinical outcomes of patients with NNRTI PDR

No	Baseline characteristics							Clinical outcomes				
	ART start date	Age	Sex	WHO stage	CD4 count	Viral load	NNRTI DRM	VL 6mo	Last known VL	Treatment outcome	Last known ART regimen	Status date
1	13.10.2014	36	Female	1	493	17,539	K103N	636	27,000	AliveOnART	3TC/ZDV/ATV/r	31.10.2019
2	07.10.2014	41	Female	2	na	84,598	K103N, V106M	40	40	AliveOnART	3TC/TDF/EFV	31.10.2019
3	13.11.2014	25	Female	2	na	86,861	Y181C	110,384	110,384	Defaulted	3TC/TDF/EFV	07.10.2015
4	11.09.2014	46	Female	1	56	na	K103N	40	40	AliveOnART	3TC/TDF/DTG	31.10.2019
5	17.09.2014	42	Male	2	123	5765	V108I, Y181C, H221Y, M184V	40	489	AliveOnART	3TC/TDF/DTG	31.10.2019
6	08.10.2014	36	Female	3	16	224,736	K103N, V138Q, Y181C	na	224,736	Died	3TC/TDF/EFV	27.01.2015
7	13.10.2014	32	Female	1	92	37,507	V179D	na	150	Defaulted	3TC/TDF/EFV	08.06.2017
8	14.10.2014	51	Male	1	na	377,623	E138K	na	377,623	Defaulted	3TC/TDF/EFV	12.01.2015
9	05.11.2014	33	Female	3	na	121,761	K103N	na	121,761	Died	3TC/TDF/EFV	06.12.2014
10	11.11.2014	42	Male	1	380	35,529	V108I, Y181C, H221Y	40	40	AliveOnART	3TC/TDF/EFV	31.10.2019
11	20.11.2014	60	Male	3	49	800,810	Y181C, H221Y	40	40	Defaulted	3TC/ZDV/ATV/r	10.09.2019
12	02.12.2014	31	Male	1	294	8117	K103N	40	40	Defaulted	3TC/TDF/EFV	11.03.2016
13	15.05.2015	32	Female	1	477	16,293	K238T	40	40	AliveOnART	3TC/TDF/DTG	31.10.2019
14	06.05.2015	25	Female	1	456	14,946	K103N, A98G, V108I	40	40	Defaulted	3TC/TDF/EFV	22.12.2018
15	28.05.2015	23	Female	2	406	1,824,545	K103N	40	40	Defaulted	3TC/TDF/EFV	17.04.2017
16	11.05.2015	40	Female	1	163	45,245	E138G	40	40	AliveOnART	3TC/TDF/DTG	31.10.2019
17	10.11.2014	29	Female	2	183	708,905	V108I, Y181C, H221Y	na	708,905	TransferOut	3TC/TDF/EFV	13.05.2015
18	18.05.2015	34	Female	1	44	204,902	E138G	7936	150	Defaulted	3TC/ZDV/ATV/r	27.05.2018
19	21.05.2015	22	Female	1	87	243,509	V106M, V179D	na	243,509	Defaulted	3TC/TDF/EFV	16.09.2015
20	20.05.2015	28	Female	3	na	329,391	G190A	na	40	TransferOut	3TC/TDF/EFV	19.03.2017
21	22.05.2015	30	Male	1	376	507,147	K103N	40	40	AliveOnART	3TC/TDF/DTG	31.10.2019
22	28.05.2015	29	Female	1	230	2,091,728	A98G, E138A	40	150	AliveOnART	3TC/TDF/DTG	31.10.2019
23	27.05.2015	25	Female	1	411	1,546,355	K103S, G190A	40	40	Defaulted	3TC/TDF/EFV	01.09.2019
24	01.06.2015	26	Female	1	209	990,009	K103N	na	990,009	Defaulted	3TC/TDF/EFV	03.11.2015
25	04.06.2015	43	Male	1	294	529,513	Y181C	na	40	AliveOnART	3TC/TDF/DTG	31.10.2019
26	23.06.2015	28	Female	1	484	na	K101E, E138A	40	40	AliveOnART	3TC/TDF/DTG	31.10.2019
27	19.06.2015	45	Female	4	179	na	K103N, A98G	na	na	Defaulted	3TC/TDF/EFV	22.05.2016

*No* Patient number, *ART* Antiretroviral therapy, *WHO stage* World Health Organization stage of clinical HIV illness, *CD4 count* Number of CD-4 positive T-cells per µl, *VL* Viral Load, Number of copies of HIV-1 RNA per ml of Serum, *NNRTI* Non-nucleoside reverse-transcriptase inhibitor, *DRM* Drug resistance mutations, *EFV* Efavirenz, *3TC* Lamivudine, *TDF* Tenofovir disoproxil fumarate, *ZDV* Zidovudine, *ATV/r* Atazanavir + Ritonavir, *DTG* Dolutegravir

## Discussion

We analyzed baseline samples from 197 participants consecutively enrolled in the LighTen cohort study. The prevalence of treatment relevant PDR in our sample reached 11.2%, almost exclusively affecting the NNRTI class. Since the Malawian HIV treatment guideline currently does not include resistance testing for ART-naïve patients prior to ART initiation, all patients initially received the standard first-line treatment of 3TC/TDF/EFV.

Although the HIVDR testing group differed significantly from the overall LighTen cohort in some baseline variables and treatment outcomes, these differences do not suggest a significant bias that would affect the level and pattern of PDR. There was a higher proportion of clients in earlier stages of HIV disease (lower WHO stage, higher viral load) in the HIVDR testing group.

The results of HIVDR testing could not influence the choice of treatment, as testing was performed retrospectively from stored samples. Among the 12 patients with K103N and/or V106M mutations (leading to a functional dual NRTI-therapy), only four were alive and on ART at the end of follow-up. Remarkably, two of these four patients were still on first-line treatment and virally suppressed. Our findings are in line with the multi-centre cohort study by Hamers et al., which found an odds ratio of 2.13 for virological failure in patients with PDR to at least one prescribed drug [12].

Our results echo other data from the region. In a cohort of Malawians living with HIV, Rutstein et al. reported the same proportion of 11% NNRTI-PDR among 46 acutely infected persons in Malawi, with a similar distribution of different sub-types of mutations [13].

According to recent data from the Malawian population-based HIV impact assessment consortium, the overall level of viral suppression in Malawi is 89%, with considerable variation between different regions in Malawi [14] For the central region, where this study was conducted, the data report proportions of treated patients with suppressed viral load between 64.9% (Lilongwe City) and 70.6% (Central West Region). [14] Our data raise the question whether differences in virological outcomes may be associated with different regional levels of PDR. Fortunately, all HIV-1 isolates analyzed here expressed phenotypes predicted to be sensitive to Malawi’s second-line treatment options.

Our observations add to the body of HIV-1 drug resistance data from Southern Africa and are in line with other reports from the region [15, 16]. The number of observations is higher than previous results from Sub-Saharan Africa, and our results include treatment outcomes of the patients.

## Conclusion

The prevalence of NNRTI-PDR was above the critical level of 10% suggested by the Global Action Plan on HIV Drug Resistance [16]. The study findings support the introduction of integrase inhibitors (i.e. dolutegravir) [17] in first-line treatment in Malawi. Furthermore, they underline the need for continued resistance surveillance and pharmacovigilance in Sub-Saharan Africa.

## Data Availability

Since the presented results are only part of the full study (see ClinicalTrials.gov NCT02381275), data can be made available upon specific request.

## Abbreviations

3TC: Lamivudine
ART: Antiretroviral therapy
DTG: Dolutegravir
DRM: Drug resistance mutation
EFV: Efavirenz
HIVDR: HIV drug resistance
LighTen: Lighthouse Tenofovir Cohort Study
NNRTI: Non-nucleoside reverse transcriptase inhibitor
NRTI: Nucleoside/nucleotide reverse transcriptase inhibitors
PDR: Pretreatment drug resistance
TDF: Tenofovir disoproxil fumarate
WHO: World Health Organization
